# Age-related differences in liver damage and inflammation after femoral osteotomy

**DOI:** 10.1007/s11357-026-02404-7

**Published:** 2026-07-29

**Authors:** Helen Rinderknecht, Rui Ding, Tong Wang, Alessa Wagner, Jasmin Maria Bülow, Nils Becker, Kernt Köhler, Katrin Bundkirchen, Claudia Neunaber, Borna Relja

**Affiliations:** 1 Department of Trauma, Hand, Plastic and Reconstructive Surgery, Ulm University Medical Center, Translational and Experimental Trauma Research, Ulm, Germany; 2 Institute of Veterinary Pathology, Justus Liebig University Giessen, Giessen, Germany; 3 Department of Trauma Surgery, Hannover Medical School, Hannover, Germany

**Keywords:** Femoral fracture, Aging, Liver inflammation, Neutrophil infiltration, RAGE, SIRT1/SIRT3, JNK

## Abstract

Fractures of long bones such as the femur are a common and serious health concern in the elderly, triggering immune responses essential for healing but also affecting remote organs like the liver. With age, the risk of fractures and immune imbalance increases, raising the likelihood of organ damage, infections, and mortality. To better understand age-dependent hepatic responses to bone injury, this study investigates early immune responses in the liver following femoral osteotomy, used here as a reproducible model of fracture. In a mouse model, young (17–26 weeks old) and aged (64–72 weeks old) male C57BL/6J mice received a femoral osteotomy with external fixation (Fx) or a corresponding sham procedure. After 24 h, inflammation, apoptosis, tissue damage, and immune responsiveness of the liver were analyzed. Aged sham animals exhibited a higher inflammatory state in sham-operated animals (neutrophil infiltration, tumor necrosis factor (TNF), interleukin (IL)-1b, chemokine (C-X-C motif) ligand 1 (CXCL1)), without corresponding increases in caspase-3-positive cells, activation of c-Jun N-terminal kinase (JNK), expression of sirtuin (SIRT) 1 or 3, or the receptor for advanced glycation end products (RAGE). Fx did not increase liver damage in young mice but showed a trend toward greater damage in aged mice. Fx significantly increased hepatic neutrophil infiltration and CXCL1 as well as TNF concentrations in both age groups. Aged mice showed weaker activation of pro- and anti-inflammatory signaling pathways after osteotomy, with reduced RAGE expression, JNK activation, and less pronounced induction of SIRT1 and SIRT3. In summary, femur osteotomy induced liver inflammation in both young and aged animals; however, older animals exhibit higher apoptosis and a superimposed inflammatory response on top of already elevated baseline inflammation in sham conditions. Further, they did not adequately activate key regulators like RAGE, SIRT1, and JNK, which coordinate inflammation and repair. Observed dysregulations may underlie the increased vulnerability to post-traumatic complications in the elderly. Understanding these age-related deficits is essential to improving therapeutic strategies.

**Graphical abstract:**

**Figure:**
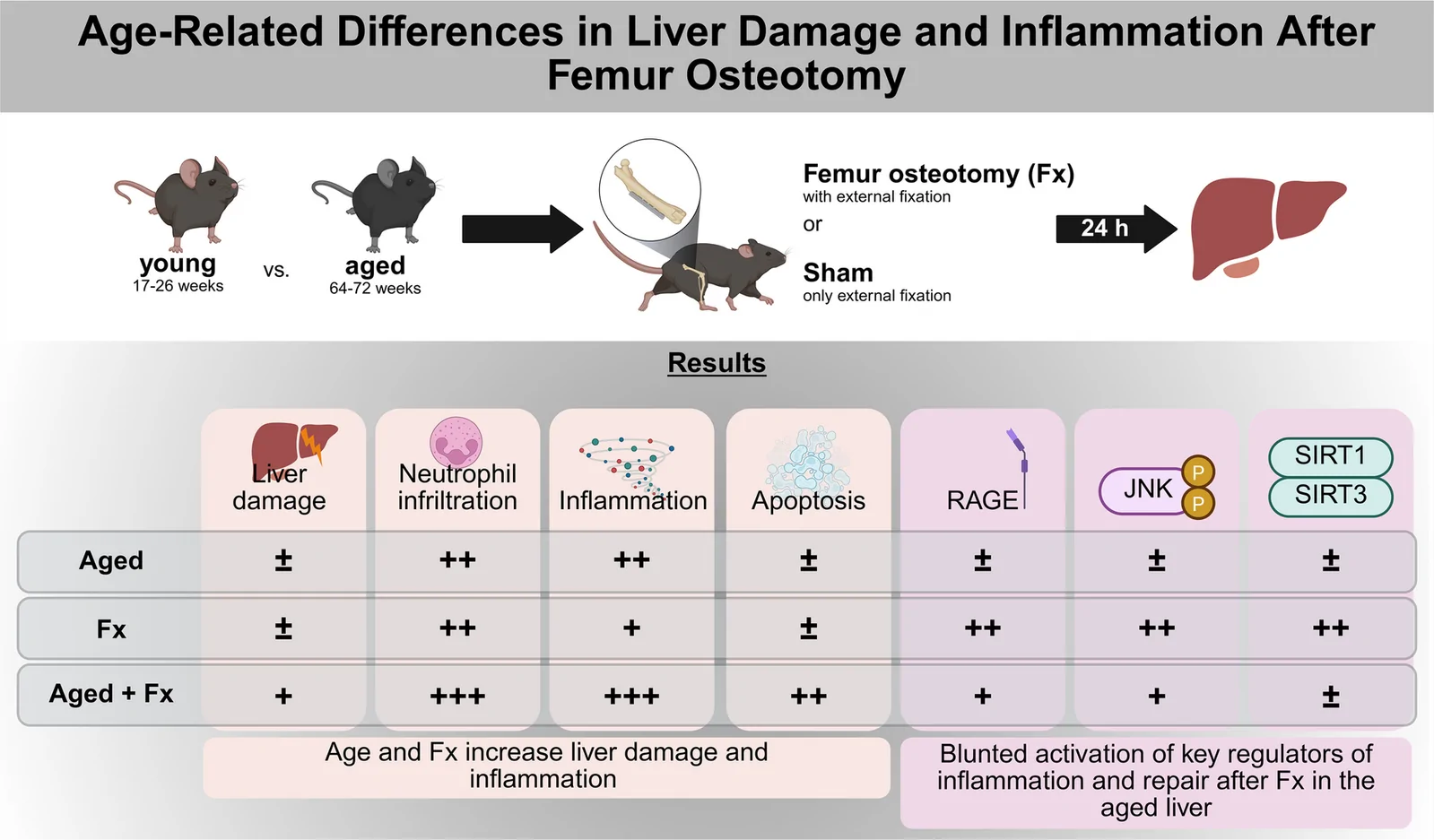

## Introduction

The global aging population is steadily increasing. According to the World Health Organization, the proportion of individuals aged 60 and older is projected to nearly double from 12% in 2015 to 22% by 2050 [1]. This demographic shift poses significant challenges to healthcare systems worldwide, and particularly understanding the physiological changes associated with aging is essential in the management of age-associated diseases and trauma. Among these, bone fractures, which rank among the most frequent injuries seen in emergency departments globally, represent a significant clinical concern often necessitating prolonged hospitalization and rehabilitation [2, 3].

Aging is accompanied by widespread physiological changes that adversely affect both the immediate immune response to injury and subsequent repair and recovery processes. A key feature of the aging process is the increased baseline inflammatory status observed in elderly individuals, which is commonly referred to as “inflammaging” [4]. This chronic low-grade inflammation is associated with an elevated risk of age-related conditions such as type 2 diabetes, atherosclerosis, and osteoporosis [5–7]. In parallel, the aging immune system undergoes profound remodeling, known as “immunosenescence,” which is characterized by a functional decline in both the innate and adaptive arms of the immune system. Age-related changes in T and B cell production and maturation, along with altered activation and responsiveness of macrophages, collectively result in a comprised ability to mount and resolve immune responses efficiently [8–12].

Advanced age is well-established risk factor for bone fractures, especially of the long bones such as the femur [13, 14]. For example, data from Germany indicate an increase in femoral fractures by 23% to 38% between 2009 and 2019, with the steepest rise in individuals over 60 years of age [14]. Such fractures have profound impact on the patients’ quality of life and contribute to increased morbidity and mortality [15–17]. Geriatric patients are especially vulnerable with 1-year mortality rates reaching 20–28% following injury, highlighting the consequences of these fractures in older populations [18].

Traumatic injuries such as femoral fractures elicit not only vascular and bony damage but a systemic inflammatory responses that extend beyond the site of injury [19]. Besides their anatomical distance, the skeletal system is tightly connected to the liver through endocrine and inflammatory signaling networks influencing bone homeostasis as well as hepatic metabolism, regeneration, and fibrosis. This bidirectional bone–liver crosstalk, mediated by circulating factors such as hepatokines and osteokines, is detrimental when chronically dysregulated and may also contribute to hepatic inflammatory changes and tissue damage following acute insults such as fractures [20]. As a central metabolic and immunological organ, the liver is essential in maintaining homeostasis through regulation of the energy metabolism, immune responses, and detoxification processes. Following injury, the acute-phase response in the liver can result in transient inflammation, oxidative stress, and tissue damage [21, 22]. However, the severity and nature of hepatic responses may vary depending on the age of the injured individual. Although, the liver has considerable regenerative capacity, aging is a significant risk factor for both acute and chronic liver dysfunction— potentially impairing its ability to respond and recover effectively after injury [23, 24].

Though age-related vulnerability to injury is recognized, the precise molecular and cellular mechanisms that drive altered hepatic responses in the elderly following trauma remain incompletely understood. In particular, the early inflammatory and apoptotic processes that occur in the liver after fracture, and how these processes differ between young and aged individuals, are not well characterized. Various pathways contribute to the regulation and resolution of post-traumatic inflammation. Damage-associated molecular patterns (DAMPs) released after injury activate receptors such as the receptor for advanced glycation end products (RAGE), which in turn stimulate nuclear factor kappa-light-chain-enhancer of activated B cells (NF-κB) signaling boosting post-traumatic inflammation [25]. Similarly, the stress-activated mitogen-activated protein kinase (MAPK) c-Jun N-terminal kinase (JNK) participates in inflammatory responses, with phosphorylation initiating downstream signaling events including apoptosis [26, 27]. Counterbalancing these pro-inflammatory pathways, the nicotinamide adenine dinucleotide (NAD⁺)-dependent deacetylases sirtuin (SIRT) 1 and SIRT3 mitigate cellular stress responses and maintain homeostasis by dampening inflammation, with SIRT3 acting as a mitochondrial checkpoint [28, 29]. A balanced response of both pro-inflammatory and regenerative pathways is necessary to effectively control inflammation and promote tissue recovery.

In this study, we examine the hepatic response to femoral osteotomy with external fixation, as a model for fracture, in young and aged mice. Within the first 24 h post-injury, we analyzed structural liver damage, cytokine expression, immune cell infiltration, and key molecular regulators of inflammation and apoptosis. By elucidating age-related differences in hepatic immune and stress responses, the study aims to provide insight into the mechanisms underlying increased susceptibility to post-traumatic complications in the elderly and enable the development of age-tailored treatment strategies.

## Methods

### Animal care

The study was authorized by the local institutional animal care and research advisory committee and the local government of Lower Saxony, Germany (approval number: 33.12–42502-04–17/2491). Male C57BL/6J mice (Janvier Labs, Le Genest-Saint-Isle, France) were housed under standard conditions in individual Type IIL cages with standard softwood as litter material. Cages, bedding, and drinking water were changed on a regular basis as described previously [21].

### Group assignment and surgical procedures

Mice aged 17–26 weeks were assigned to the young group, while those aged 64–72 weeks comprised the old group. A total of 12 animals per age group were randomly allocated to undergo either sham operations or fracture-mimicking osteotomy (Fx) procedures, resulting in final group sizes of six animals each (*n* = 6).

All surgical procedures were performed under deep isoflurane inhalation anesthesia (Baxter Deutschland GmbH, Unterschleißheim, Germany), with surgeries commencing only after confirmation of absent interdigital reflexes. Throughout the operations, mice were maintained on heating pads to preserve their body temperature. Analgesia was provided via subcutaneous injections of carprofen (5 mg/kg body weight; Rimadyl®, Zoetis Deutschland GmbH, Berlin, Germany) and butorphanol (1 mg/kg body weight; Torbugesic®, Zoetis Deutschland GmbH), along with local anesthesia at the surgical site using prilocaine hydrochloride. Surgical procedures were performed as described before [21, 30]. In brief, an external fixator (MouseExFix simple L 100%, RISystem, Davos, Switzerland) was mounted on the right femur of all animals according to the manufacturer’s guidelines, requiring a skin incision and preparation of the femur in all animas. In Fx animals, a diaphyseal osteotomy was performed between the two central pins of the fixator using a 0.44-mm Gigli wire saw (RISystem), representing the only additional procedure compared to sham animals. Wounds in both experimental groups were closed using Prolene 6-0 sutures (Ethicon, Cincinnati, USA). Until fully awake, animals were kept under red light and subsequently housed in individual cages. After surgery, animals were permitted unrestricted movement and metamizole was added to the drinking water (200 mg/kg body weight) for pain management. Throughout the study, animals were routinely evaluated for their activity, overall health, posture, pain symptoms, and any signs of lameness.

### Sacrifice and harvesting

Animals were sacrificed with ketamine (75 mg/kg body weight) and medetomidine (1 mg/kg body weight) injected intraperitoneal 24 h after surgery. Following the absence of the interdigital reflex, a midline laparotomy was conducted to expose the abdominal cavity. Cardiac blood was collected using a 25-gauge needle, and euthanasia was subsequently completed via cervical dislocation. Collected blood samples were centrifuged at 7,000 rpm for 5 minutes at room temperature to separate plasma, which was then stored at −80 °C until further use. To access the thoracic cavity, the incision was extended along the chest wall. Mice were perfused via the heart with 20 mL of PBS using a 21-gauge blunt-tipped syringe (BD, Franklin Lakes, USA). Following perfusion, the left lateral lobe of the liver was dissected, snap frozen in liquid nitrogen, and stored at −80 °C for further analysis. In the following, mice were perfused with 4% buffered zinc-formalin *(*Thermo Fisher Scientific, Waltham, USA). The right liver lobe was ligated and fixed in the same fixative overnight for histological analysis.

### Histological analysis

Liver lobes, fixed in 4% buffered zinc-formalin (Thermo Fisher Scientific) overnight, were embedded in paraffin. Paraffin blocks were then sectioned into 3 µm slices. The sections were deparaffinized with Roti Histol (Carl Roth, Karlsruhe, Germany) and rehydrated through a descending ethanol series in preparation for subsequent histological analysis.

#### Evaluation of liver damage

Liver damage was assessed using hematoxylin/eosin (H&E)-stained sections. First, the sections were stained with hematoxylin solution (Carl Roth) for 10 min at room temperature. Then, they were rinsed with water for 10 min and counterstained with eosin solution (Carl Roth) for additional 3 min. Using an ascending ethanol series, the sections were dehydrated and then mounted using Mountex (Medite Medical GmbH, Burgdorf, Germany). The liver pathology was evaluated by an independent veterinary pathologist. In a blinded manner, the parameters random necrosis, single-cell degeneration, or necrosis (including individualization), zonal (perivenous) necrosis, cellular vacuolization, and vacuolization of hepatocytes were scored individually for each section on a semi-quantitative grading scale: 0 (not observed), 1 (mild), 2 (moderate) or 3 (marked). The liver injury score (LIS) was calculated as the mean of all individual parameter scores for each specimen [31].

#### Immunohistochemistry

According to the manufacturer’s instructions, epitopes were recovered by heat using R-Universal Epitope Recovery Buffer (Aptum, Kassel, Germany) in the 2100-Retriever (Prestige Medical, Blackburn, England) at 121 °C for 20 min. To block endogenous oxidases, a hydrogen peroxide block (Peroxidase UltraVision Block, Thermo Fisher Scientific) was performed for 20 min. Primary antibodies against active caspase-3 (Asp175, #9661, Cell Signaling Technology, USA, 1:300), neutrophil elastase (NE, bs-6982R, Bioss, Woburn, MA, USA, 1:200), RAGE (ab3611, Abcam, Cambridge, UK, 1:100), SIRT1 (ab189494, Abcam, 1:500), and SIRT 3 (SAB1301489, Sigma-Aldrich, St. Louis, MO, USA, 1:100) were applied according to the manufacturer’s instructions. Sections were incubated with primary antibodies diluted in Antibody Dilution Buffer (Dako Cytomation) for 1 h at room temperature. This was followed by a 30-min incubation at room temperature with an HRP-conjugated secondary antibody (Histofine Simple Stain Mouse MAX PO (R), Nichirei Biosciences Inc, 414311F). Specific antibody binding and immunoreactivity were detected using the chromogenic substrate 3-amino-9-ethylcarbazole (AEC; DCS Innovative Diagnostik-Systeme, Hamburg, Germany). Hematoxylin (Carl Roth, Karlsruhe, Germany) was used to counterstain the cell nuclei. Slides were mounted and micrographs were recorded in ×40 magnification with an Axio Observer Z1 microscope (×40 objective, Carl Zeiss, Göttingen, Germany). Images were evaluated using ImageJ software. For quantification, NE- and caspase-3-positive cells were quantified across 25 randomly chosen high-power fields at ×400 magnification. The red chromogenic signal was quantified across the entire digital slide. After staining with AEC, whole-slide images were acquired, and the complete tissue area was analyzed without selecting regions of interest. The expression levels of RAGE, SIRT1, and SIRT3 were assessed by calculating the mean pixel intensity values of the red signal for the full slide, providing an unbiased measure of overall immunoreactivity with the Zen Pro software (version 3.2, Carl Zeiss).

### Dry chemistry testing

Serum levels of liver damage markers total protein, glutamate-oxaloacetate transaminase (GOT), and lactate dehydrogenase (LDH) were quantified using the Arkray Spotchem EZ SP-4430 system according to the manufacturer's instructions.

### Enzyme-linked immunosorbent assay

Liver tissue was homogenized in ice-cold lysis buffer (FNN0021, Invitrogen™, Thermo Fisher Scientific) and centrifuged at 20,000 × g for 30 min. The debris-free supernatant was stored at −80 °C until further analysis. The concentrations of protein isolates were determined using the Pierce™ BCA Protein Assay Kit (Thermo Fisher Scientific), following the manufacturer’s instructions. Cytokine levels of tumor necrosis factor (TNF, former TNF-α), interleukin (IL)−1β, IL-6, and chemokine (C-X-C motif) ligand 1 (CXCL-1) were quantified using murine ELISA kits (R&D Systems) following the manufacturer’s instructions in a total of 100 µg of protein per sample. Results were quantified with the Spark M10 microplate reader (Tecan, Männedorf, Switzerland).

### Gene expression analysis

After mechanical homogenization of the liver tissue using the Percellys 24 Homogenizer (Bertin Technologies, Montigny-le-Bretonneux, France), RNA from tissue lysates was isolated using the RNeasy mini kit (Qiagen, Hilden, Germany). The quantity and quality of obtained RNA were assessed spectrophotometrically. Following the manufacturer’s instructions, cDNA was synthesized with the iScript™ cDNA Synthesis Kit (BioRad, Hercules, USA). Reverse transcription-quantitative PCR (RT-qPCR) reactions were set up in a final volume of 25 µL using SYBR Green qPCR Master Mix (Bio-Rad), according to the manufacturer’s instructions. Amplification was performed on a CFX96 Touch Real-Time PCR Detection System (Bio-Rad). Expression of target genes *Tnf* (qMmuCED0004141; BioRad), *Cxcl1* (qMmuCED0047655; BioRad), *Il-6* (qMmuCID0005613; BioRad), and *Il-1b* (qMmuCEP0054181; BioRad) were quantified relative to the housekeeping gene *Gapdh* (glyceraldehyde 3-phosphate dehydrogenase, qMmuCED0027467; BioRad). Relative gene expression was calculated by means of the comparative threshold-cycle (CT) method (2^−ΔΔCT^).

### Western blot

Liver tissues were homogenized in lysis buffer (FNN0021, Invitrogen™) at 4 °C, and the homogenates were centrifuged at 20,000 × g for 30 min at 4 °C to remove cellular debris. The resulting supernatant was collected and stored at −80 °C until used for protein analysis. The concentrations of protein isolates were determined using the Pierce™ BCA Protein Assay Kit (Thermo Fisher Scientific), following the manufacturer’s instructions. Proteins (15 µg per sample) were separated by electrophoresis on a 10% sodium dodecyl sulfate-polyacrylamide gel and subsequently transferred to polyvinylidene difluoride membranes (Thermo Fisher Scientific). Free binding sites were blocked for 1 h with 5% bovine serum albumin (BSA; Sigma-Aldrich) in TBS-T buffer (20 mM Tris, 150 mM NaCl, 0.05% Tween, pH 7.6) at room temperature. Membranes were then incubated with primary antibodies targeting phosphor-JNK (P-JNK, 4668S, Cell Signaling, 1:1000), JNK (67096S, Cell Signaling, 1:1000) and β-actin (MAB8929, R&D Systems, 1:1000) diluted in 1 to 5 % bovine serum albumin in TBS-T buffer at 4 °C overnight. Secondary horseradish peroxidase (HRP)-conjugated antibodies, Anti-mouse IgG HRP-linked Antibody (7076P2, Cell Signaling, 1:3000) or Anti-rabbit IgG HRP-linked Antibody (7074P2, Cell Signalling, USA, 1:3000), were applied for 1 h at room temperature. Proteins were detected with ECL™ western blot detection reagent (BioRad) and captured with the ChemiDoc XRS+ system (BioRad). Protein expression was quantified via densiometric analysis using ImageJ. The JNK phosphorylation ratio was calculated after separate normalization of JNK and P-JNK expression to reference protein β-actin. The data were further normalized to the young sham group.

### Statistical analysis

For visualization and statistical analysis, GraphPad Prism 10 software (GraphPad Software, Inc., San Diego, CA) was used. Normality of data was assessed using Shapiro-Wilk tests. Differences between groups were analyzed using two-way ANOVA, followed by Tukey’s Honestly Significant Difference post hoc test for all pairwise comparisons. Data are presented as bar graphs showing the mean + standard error of the mean. Statistical significance was defined as * *p* < 0.05.

## Results

### Age-dependent hepatic apoptosis, tissue damage, and neutrophil infiltration following femoral osteotomy

**Fig. 1:**
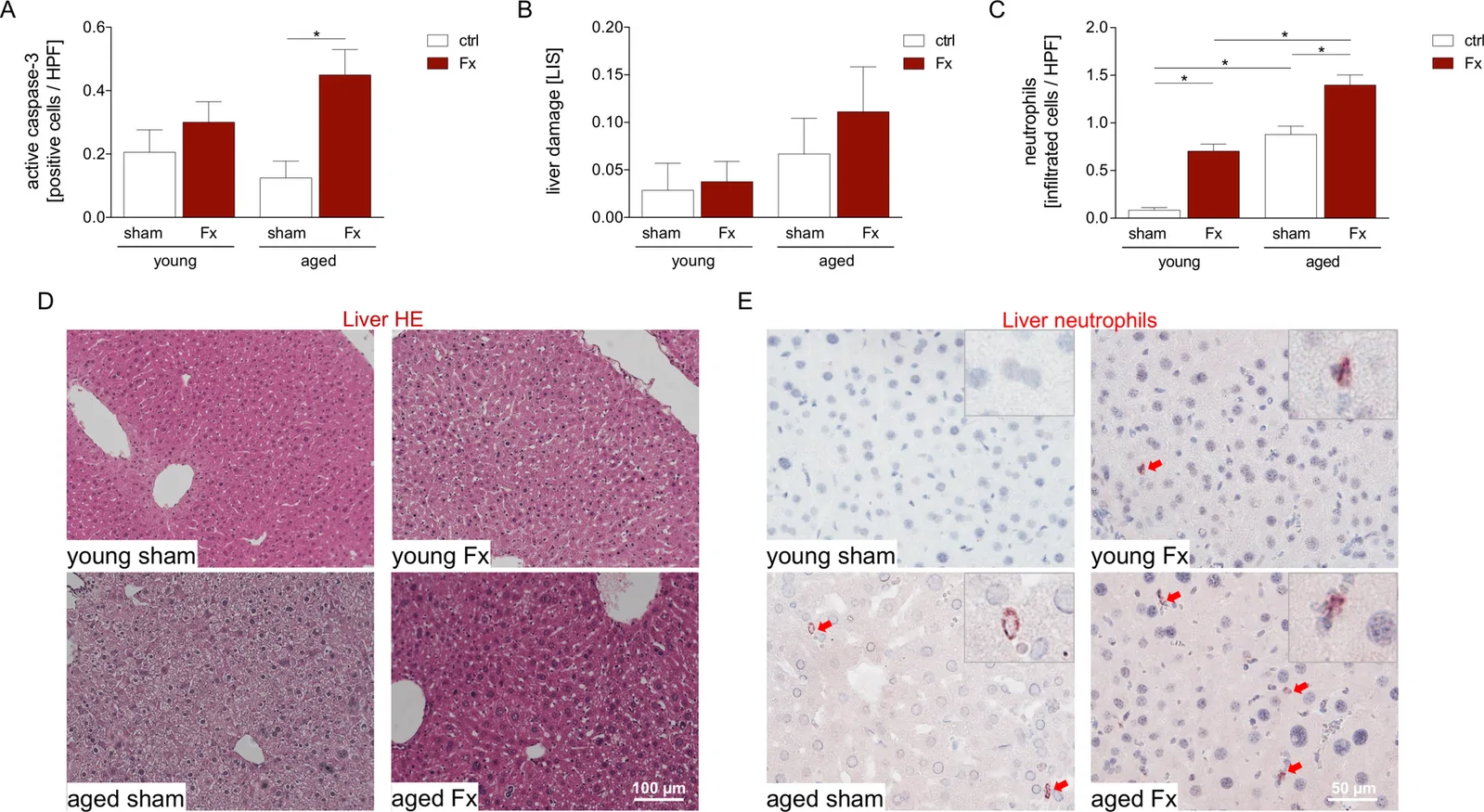
Hepatic apoptosis, tissue damage, and neutrophil infiltration following femoral osteotomy (Fx) or sham procedures in young and aged mice. Mice aged 17 to 24 weeks (young) or 64 to 72 weeks (old) (*n* = 12 each) were randomly assigned to undergo either femoral osteotomy with external fixation (Fx) or a sham surgery with only external fixation. Sampling and analyses were carried out 24 h after the procedures. **A** Quantification of caspase 3-positive cells. **B** Liver injury score (LIS): Liver injury parameters (random necrosis, single-cell degeneration/necrosis, zonal necrosis, and vacuolization) were scored blinded on a scale from 0 (not observed) to 3 (marked). The LIS was calculated as the mean of all parameter scores per specimen. **C** Neutrophil infiltration quantified from neutrophil elastase-stained sections. **D** Representative micrographs of hematoxylin and eosin (H&E)-stained liver sections at × 40 magnification. **E** Representative micrographs of neutrophil in representative liver sections at × 40 magnification. Small red arrows highlight the infiltrated neutrophils. Data are presented as mean + standard error of the mean; *n* = 6 per group

Young and aged mice underwent either femoral osteotomy stabilized with an external fixator (Fx) or a sham procedure involving only mounting of an external fixator without osteotomy. Liver tissues were collected 24 hours post-procedure for analysis. Fx significantly increased the number of caspase-3-positive cells, a marker for apoptosis, in aged mice compared to their sham controls, as shown in Fig. 1A. In contrast, young Fx mice did not exhibit increased caspase-3-positive cells relative to their sham counterparts, and no significant differences were observed between the young and aged sham or Fx groups. Liver injury was assessed using the LIS. Although no statistically significant differences were detected across groups, aged mice, particularly those subjected to Fx, displayed a trend toward increased liver damage (*p*=0.1351) (Fig. 1B and D). Given the key role of neutrophils as early mediators of inflammation after tissue injury, their hepatic infiltration was examined. Aged animals exhibited significantly higher neutrophil counts compared to young mice under both sham and Fx conditions. Moreover, Fx caused a marked increase in hepatic neutrophil infiltration in both age groups. Notably, neutrophil levels in young Fx mice approximated those observed in aged sham mice (Fig. 1C and E).

In summary, aged mice exhibited heightened hepatic apoptosis and neutrophil infiltration following Fx, along with a trend toward more pronounced liver injury.

### Age-dependent changes in circulating hepatic injury markers

**Fig. 2:**
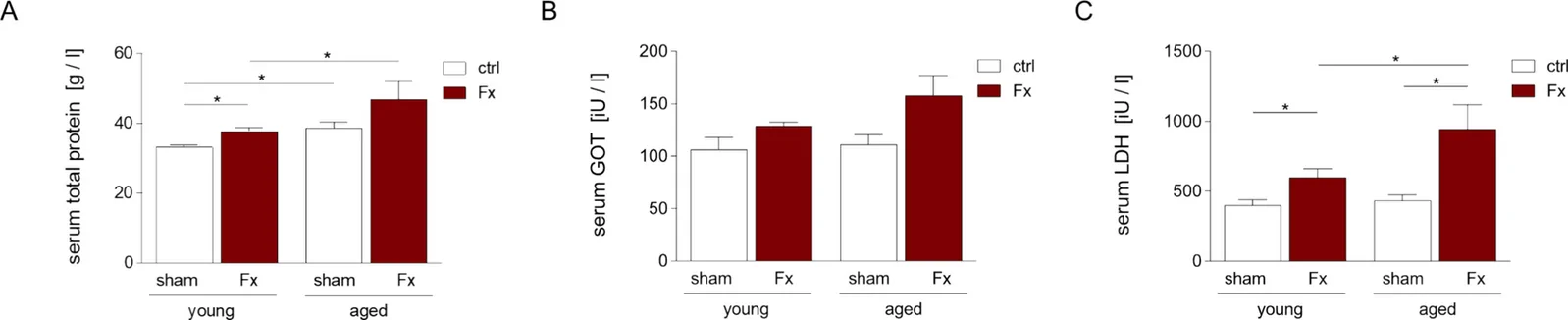
Circulating hepatic injury markers serum total protein, serum glutamate–oxaloacetate transaminase (GOT), and serum lactate dehydrogenase (LDH) following femoral osteotomy (Fx) or sham procedures in young and aged mice. Mice aged 17 to 24 weeks (young) or 64 to 72 weeks (old) (*n* = 12 each) were randomly assigned to undergo either femoral osteotomy with external fixation (Fx) or a sham surgery with only external fixation. Sampling and analyses were carried out 24 h after the procedures. **A** Serum total protein in g/l. **B** Serum GOT in iU/l. **C** Serum LDH in iU/l. Data are presented as mean + standard error of the mean; *n* = 6 per group

Hepatic injury markers were assessed in circulation. Results are displayed in Fig. 2. Aged sham mice exhibited a significant increase in serum total protein compared to young sham animals, and this difference persisted after Fx (Fig. 2A). Following Fx, young mice displayed a significant elevation in total protein, whereas aged mice showed a similar trend that did not reach statistical significance (*p *= 0.1856) (Fig. 2A). Serum GOT and LDH levels did not differ significantly between young and aged sham cohorts. Fx induced a significant increase in serum LDH in both age groups, with aged Fx mice showing higher LDH levels post-injury compared to young Fx animals (Fig. 2C). Serum GOT levels followed a similar pattern, increasing after Fx and tending to be higher in aged animals, although these changes did not reach statistical significance (*p *= 0.0649) (Fig. 2B). In summary, aged mice exhibited elevated circulating hepatic injury markers following Fx.

### Age-dependent hepatic pro-inflammatory cytokine response after femoral osteotomy

**Fig. 3:**
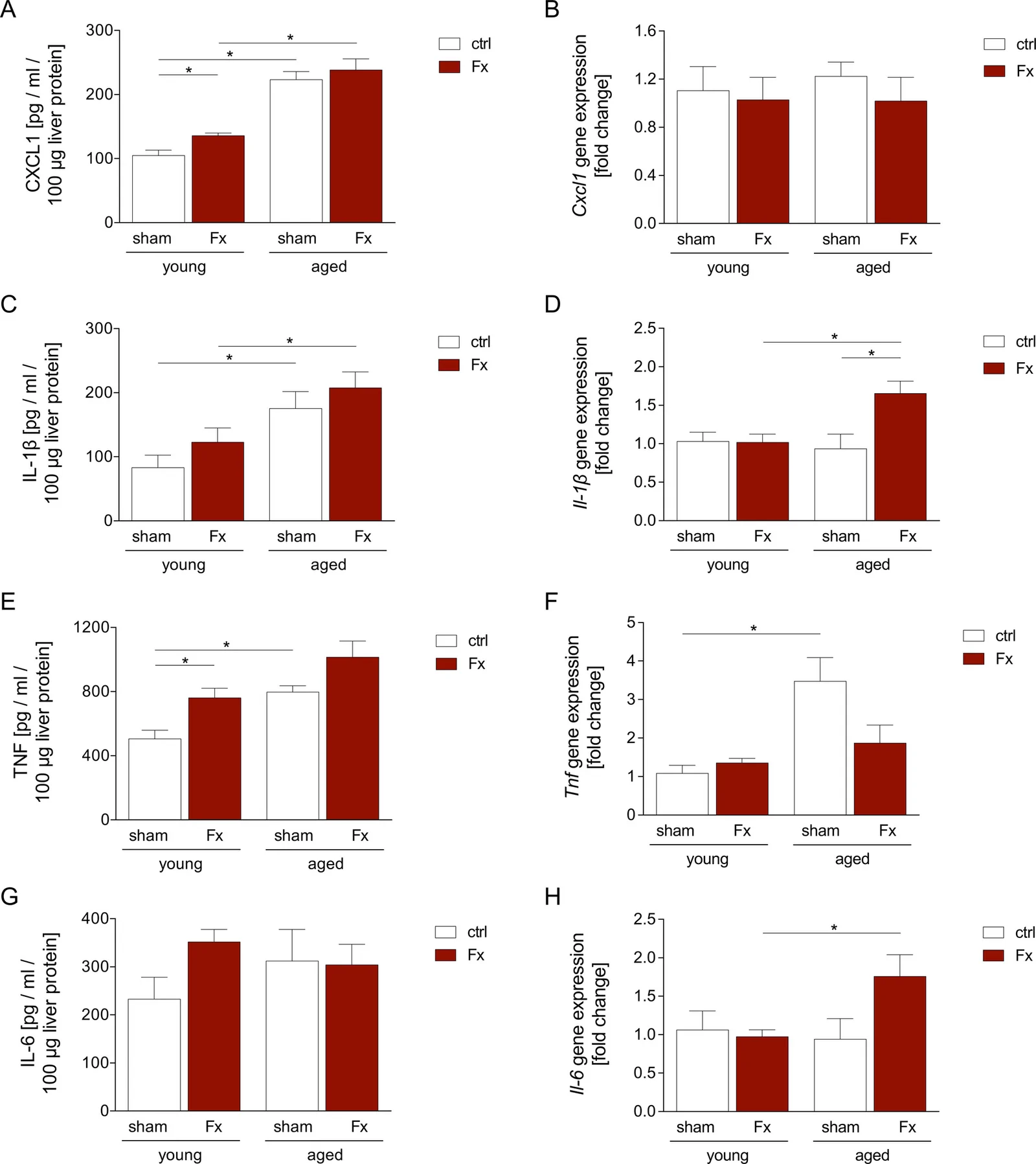
Hepatic expression of pro-inflammatory cytokines in young and aged mice following femoral osteotomy (Fx) or sham procedures. Mice aged 17 to 24 weeks (young) or 64 to 72 weeks (old) (*n* = 12 each) were randomly assigned to undergo either femoral osteotomy with external fixation (Fx) or a sham surgery with only external fixation. Sampling and analyses were carried out 24 h after the procedures. **A**, **C**, **E**, **G** Protein levels of chemokine (C-X-C motif) ligand 1 (CXCL1) (**A**), interleukin (IL)−1β (**C**), tumor necrosis factor (TNF) (**E**), and IL-6 (**G**) in pg/mL out of 100 µg total liver protein. **B**, **D**, **F**, **H** Gene expression of *Cxcl1* (**B**), *Il1b* (**D**), *Tnf* (**F**), and *Il6* (**H**). Relative gene expression was analyzed using the comparative threshold cycle (ΔΔCt) method (2^^−^ΔΔCt) after normalization to *Gapdh* as a housekeeping gene. Sham young animals served as the baseline reference group. Data are presented as fold change relative to gene expression in sham young animals. Data are presented as mean + standard error of the mean; *n* = 6 per group

Hepatic inflammation was assessed by measuring both gene expression and protein levels of key pro-inflammatory cytokines TNF, IL-1β, and IL-6 and chemokine CXCL-1, as shown in Fig. 3. At baseline, aged sham mice showed a significant increase in CXCL-1, IL-1β, and TNF protein levels compared to young sham animals, while IL-6 protein showed a non-significant trend (*p *= 0.5368) toward elevation (Fig. 3A, C, E and G). Fx induced a significant increase in CXCL-1 protein levels in young mice, but not in aged animals—a pattern not reflected at the mRNA level (Fig. 3A and B). IL-1β protein levels showed a non-significant upward trend in both age groups after Fx (*p *= 0.5887 and *p *= 0.3290, respectively); however, *Il1β* mRNA levels increased significantly in aged Fx mice compared to aged sham controls and young Fx animals (Fig. 3C and D). TNF protein and mRNA levels were significantly elevated in sham-aged mice compared to young sham mice. After osteotomy (Fx), TNF protein levels tended to increase in both age groups, reaching statistical significance only in the young group. A similar trend was observed for TNF gene expression in young animals, whereas aged animals exhibited a non-significant (*p *= 0.0667) opposite tendency (Fig. 3E and F). No significant changes in IL-6 protein levels were detected between age groups or in response to Fx (Fig. 3G). Nonetheless, *IL6* gene expression was significantly upregulated in aged Fx mice compared to young Fx controls (Fig. 3H). In summary, aged mice exhibited a heightened baseline inflammatory state in the liver. Femoral osteotomy amplified hepatic inflammation in both age groups, with young mice showing more robust protein-level changes, while aged mice exhibited selective increases at the transcriptional level.

### Age-dependent hepatic susceptibility to react to inflammatory stimuli and anti-inflammatory signaling after femoral osteotomy

**Fig. 4:**
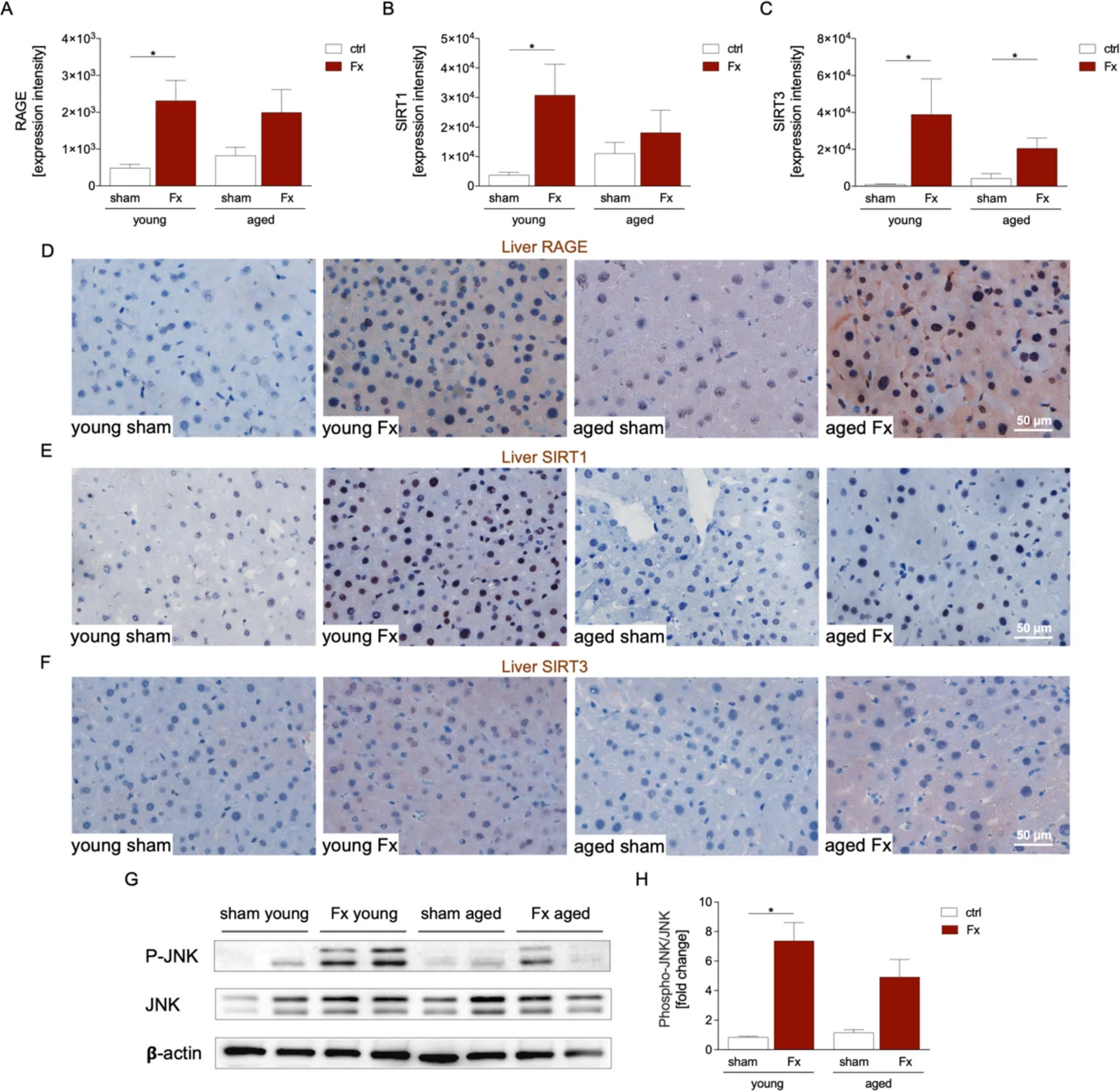
Expression of receptor for advanced glycation end products (RAGE), sirtuins (SIRT1/3), and activation of the c-Jun N-terminal kinase (JNK) pathway in liver tissue following femoral osteotomy (Fx) or sham procedures in young and aged mice. Mice aged 17 to 24 weeks (young) or 64 to 72 weeks (old) (*n* = 12 each) were randomly assigned to undergo either femoral osteotomy with external fixation (Fx) or a sham surgery with only external fixation. Sampling and analyses were carried out 24 h after the procedures. **A**–**C** Expression intensities of RAGE (**A**), SIRT1 (**B**), and SIRT3 (**C**). **D**–**F** Exemplary micrographs of RAGE (**D**), SIRT1 (**E**), and SIRT3 (**F**) stained liver sections recorded in × 40 magnification. Red signal intensity, that corresponds to positive antibody binding, was quantified in defined regions of interest; non-tissue areas and artifacts were excluded, background substracted, and then, the mean signal intensity within these regions, which limits the impact of differences in cell number and background staining was calculated. **G** Exemplary images of P-JNK, JNK, and β-actin protein analysis by western blot showing the protein levels in the livers of two separate animals per condition. **H** JNK phosphorylation ratio (P-JNK/JNK) expressed as fold change relative to young sham animals. P-JNK and JNK were each independently normalized to the reference protein β-actin prior to ratio calculation. Data are presented as mean + standard error of the mean; *n* = 6 per group

To assess RAGE signaling and related stress response pathways, protein expression levels of RAGE, SIRT1, SIRT3, and phosphorylated JNK (P-JNK) were quantified, as shown in Fig. 4. The expression of RAGE was comparable between young and aged animals under sham conditions or after Fx, as shown in Fig. 4A and D. However, Fx significantly upregulated RAGE expression in young mice, while aged mice showed only a non-significant trend toward increased expression (*p *= 0.0649). The expression of both sirtuins, SIRT1 and SIRT3, key regulators of anti-inflammatory and stress responses, also demonstrated age-dependent differences. In young mice, both SIRT 1 and SIRT3 were significantly upregulated following Fx (Fig. 4B, C, E and F). In contrast, in aged mice, only SIRT3 showed a significant increase after Fx, while SIRT1 levels remained unchanged. Similarly, activation of the inflammatory signaling molecule JNK, assessed via its phosphorylation (P-JNK), was significantly elevated in young Fx mice (Fig. 4G and H). In aged mice, P-JNK levels showed a modest however non-significant increase (*p *= 0.1413) in response to Fx. Baseline levels of RAGE, SIRT1, SIRT3, and P-JNK did not differ between young and aged mice under sham conditions. In summary, femoral osteotomy induced a robust upregulation of RAGE, SIRT1, SIRT3, and P-JNK signaling pathways in young mice, whereas aged mice exhibited a markedly blunted molecular response, indicating impaired activation of key regulators of inflammation and cellular stress.

## Discussion

Long bone fractures are an increasing health concern in the aging population. While their local consequences are well characterized, systemic effects, particularly on distant organs such as the liver, remain poorly understood despite known interdependencies. This study examined hepatic responses to femoral fracture-mimicking osteotomy in young and aged mice. Since sham and Fx animals underwent identical surgical procedures, including skin incision and placement of the external fixator, any observed differences between sham and THFx groups can be attributed to the additional bone injury caused by the osteotomy. The study reveals that aging is associated with higher liver inflammation in sham-operated animals, increased hepatic damage, increased apoptotic cell death, neutrophil infiltration, and impaired activation of key pro-inflammatory signaling pathways (RAGE, JNK) as well as negative regulators of inflammation (SIRTs) following additional bone injury.

Aged mice showed an elevated pro-inflammatory profile in sham-operated animals, consistent with the concept of “inflammaging,” which is widely linked to aging and impaired tissue repair [4, 32, 33]. The accumulation of senescent cells, adopting a pro-inflammatory secretory phenotype, likely contributes to a heightened inflammatory state, further promoting immune cell recruitment and explaining elevated neutrophil infiltration even without major injury [34]. Despite increased inflammation, aged sham mice showed no corresponding increase in apoptosis or liver damage including damage markers at the studied time point, suggesting that low-grade inflammation had not yet translated into tissue injury, which is in line with previous findings [31]. It must be noted that also sham animals underwent minor surgery including skin incision and mounting of the external fixator. This implies that the elevated inflammation observed in aged mice could be attributed to both age-related susceptibility and a potentially heightened response to minor injury. Femoral osteotomies amplified liver inflammation in both age groups. Aged mice showed similar but superimposed effects on their already existing inflammatory background from sham operations with partially distinct cytokine response patterns. Although most changes did not reach statistical significance, the observed pattern suggests dynamic immune regulation, with age-related differences in cytokine responses which is consistent with prior studies [35–37]. In more detail, previous research indicates that aging does not simply skew the inflammatory response in one direction, but shifts the composition of mediators in early inflammatory networks, likely reflecting inflammaging-related changes in immune cell composition, activation state, and immunosenescence [35–37]. Moreover, an overall decline in transcriptional activity with aging may also contribute to the observed differences in gene and protein expression levels between the two age groups [31, 38]. Neutrophil accumulation in distant organs may also result from altered chemokine receptor signaling, as shown in aged mice with local injury where mast cell-derived CXCL1 desensitized CXCR2 signaling in neutrophils, thereby impairing their directional motility [39].

Femoral osteotomy caused a subtle increase in liver injury and associated circulating damage markers in young mice, whereas aged mice exhibited overall more severe hepatic injury, paralleling inflammatory parameters. The lack of significant differences in the liver injury score likely reflects the scoring system’s limited sensitivity to subtle histological changes [31]. In previous work, using the same score we could demonstrate that femoral osteotomy combined with hemorrhagic shock (THFx) markedly exacerbates hepatic injury and inflammation in aged mice, associated with a partial ceiling effect in NF-κB activation [31]. This study confirms these findings, and the direct comparison reveals that the severity of the injury has a significant impact on the magnitude of liver damage. However, more severe liver damage in older animals in response to minor injuries, such as isolated osteotomy, can also have clinical implications due to reduced organ reserve and increased susceptibility to subsequent stress, likely predisposing aging livers to more pronounced deficits in the event of more severe injuries.

While increased apoptosis has mainly been reported in more severe or prolonged but young trauma models [40–42], in this study, solely aged mice exhibited significantly higher hepatocellular apoptosis post-osteotomy. This suggests reduced resilience of the aged liver, either due to higher susceptibility to trauma-induced cell death or impaired clearance of apoptotic cells, both recognized risk factors for liver injury [43, 44]. Although some studies report lower apoptosis with aging [45], in our study, low-grade inflammation and senescence likely remain compensated under milder sham conditions, with osteotomy-induced stress, however, potentially exceeding the liver’s compensatory capacity, exposing underlying defects [46, 47].

At the signaling level, RAGE, sirtuins, and JNK phosphorylation were examined, as they are implicated in inflammatory regulation and homeostasis restoration. DAMP receptor RAGE interacts with several ligands, including advanced glycation end-products (AGEs), the reduced form of high-mobility group box 1 (HMGB1), and S100β, all of which are known to accumulate under inflammatory conditions or aging [48–50]. Activation of RAGE initiates downstream signaling cascades including JAK-STAT and NF-κB pathways driving initial post-traumatic inflammation and tissue regeneration. RAGE expression was significantly upregulated in young mice following osteotomy, indicating an adequate injury response. While previous studies reported increased RAGE ligand levels in aged livers, we did not observe significantly elevated RAGE receptor expression in the aged livers [51]. Taken together with evidence that RAGE blockade attenuates liver injury in young but not aged animals, the data suggests that hepatic damage in aged mice is driven, at least in part, by RAGE-independent pathways [52, 53]. Whether RAGE plays a protective or detrimental role in remote liver injury remains context-dependent and requires further investigation [54].

Sirtuins, particularly SIRT1 and SIRT3, are key regulators of cellular stress resilience [28, 55]. SIRT1, primarily cytoplasmic, acts as a negative regulator of inflammation through deacetylation of the NF-κB p65 (RelA) subunit, thereby reducing its transcriptional activity [56, 57]. SIRT3, located in mitochondria, primarily tunes energy production and minimizes oxidative damage [58, 59]. In this study, young animals exhibited robust SIRT1 and SIRT3 upregulation after osteotomy, indicating a coordinated endogenous response to injury. In aged mice, SIRT1 failed to upregulate, and SIRT3 increased with reduced capacity. Both SIRT1 and SIRT3 reductions have been reported with aging [29, 60]. Blunted SIRT1 responses in aged mice may contribute to prolonged NF-κB activation and sustained inflammation, aligning with previous reports linking reduced SIRT1 expression to impaired regeneration after injury in aging [61, 62]. Similarly, SIRT3 as a central mitochondrial longevity factor has been shown to decline with age, and its loss is associated with increased hallmarks of senescence and accelerated fibrotic or inflammatory damage [63–65]. SIRT1 and 3 are reported to be downregulated in the initial inflammatory phase after injury (≤ 6 h); however, their upregulation guides toward a regenerative and proliferative phase (12 h to days) [66, 67]. At the time of our analysis, the simultaneous increase in SIRT1 and pro-inflammatory cytokines in young mice subjected to osteotomy likely reflects this transition in which NF-κB-driven inflammation persists while SIRT1-mediated counterregulatory mechanisms emerge and attenuate ongoing inflammatory signaling. Overall, the absence of SIRT upregulation in aged animals may contribute to delayed or insufficient regeneration and promote cellular senescence.

JNK, a stress-activated MAPK, plays a complex and context-dependent role in liver physiology, inflammation, and regeneration [26, 27]. While its acute and transient activation promotes hepatocyte proliferation and regeneration, its loss or chronic activation has been associated with liver pathologies, including fibrosis and metabolic diseases [68–70]. In young animals, osteotomy induced significant JNK activation, supporting its role in acute hepatic stress. Despite reports of enhanced JNK with aging, our data show that older mice fail to fully activate JNK post-osteotomy, reflecting impaired stress signaling and possibly delayed, compromised, or exhausted initiation of repair [71]. This blunted response may reflect altered upstream MAPK signaling, providing a mechanistic basis for insufficient JNK activation in aged livers [72].

Taken together, the observations raise the question whether the blunted injury response in aged animals reflects an absolute signaling deficit or a ceiling effect due to already elevated baseline inflammation. However, closer inspection argues against a ceiling effect. Despite elevated hepatic inflammation, aged sham animals do not show a correspondingly increased adaptive signaling response, contrasting the previously reported increase in IκB phosphorylation in aged sham mice [31]. Second, while osteotomy induces hepatic inflammation in both groups, aged animals mount a markedly reduced adaptive signaling response, indicating that induction capacity is preserved but attenuated, rather than exhausted. Together, this suggests an age-dependent deficit in adaptive signaling capacity which could also be responsible for the increased observed hepatic damage in the aged. This however warrants further detailed investigation.

This study provides insights into age-related hepatic changes following bone injuries and fractures. However, there are limitations that must be considered and the study results should be interpreted in the context of these constraints. The analysis was limited to a single time point, 24 h post-injury. This captured early stages of inflammation but neglected the initial early inflammatory events and the resolution and repair of hepatic inflammation. Future studies should include additional time points to fully characterize the temporal dynamics and could clarify whether aged mice appropriately resolve inflammation or are prone to complications. There are always inherent challenges in comparing clinical fractures with in vivo models. In this study, the employed femoral osteotomy with external fixation, a well-established open fracture model, has limitations in its clinical translation. While the osteotomy provides the reproducibility necessary for controlled in vivo experimentation, the model does not replicate typical sterile closed fractures treated with internal fixation and thus differs from standard clinical practice [73, 74]. In more detail, the model is unable to capture variability in fracture pattern, introduces additional soft tissue injury, neglects patient-related factors such as comorbidities, and can of course not capture species specificities in bone biology and healing processes. The extent to which the presented findings translate to physiological situations in humans therefore warrants further investigation and future experiments could include a sterile closed fracture model stabilized by intramedullary nailing to more closely recapitulate the clinical scenario. In this context, it should also be noted that no true uninjured baseline was included, as sham animals also underwent external fixator placement including soft tissue injury by skin incision. Previous studies have shown that similar minor sham procedures do not induce hepatic changes in young animals; however, their impact on aged animals cannot be fully excluded [75, 76]. Therefore, the findings in sham-aged animals may indicate increased hepatic vulnerability, elevated baseline inflammation, or both. Nevertheless, the chosen study design ensures that any differences observed between sham and Fx animals can be specifically attributed to the bone damage caused by the osteotomy, rather than to the surgical interventions themselves. Furthermore, the inability of the aged animals to mount an appropriate adaptive signaling response can be concluded without comparison to naïve control animals. To conclude, future studies should include pre-sham aged animals to establish a true baseline and further clarify whether the observed hepatic changes in the sham animals reflect age-related vulnerability, the effects of the sham procedure, or both. The presented studie does not take sex-specific differences into account as it exclusively focused on male mice, which limits the generalizability of the findings to females. This distinction is of particular relevance given that postmenopausal hormonal changes and reduced bone mineral density place elderly women at particularly increased risk of fractures and associated complications [77]. To improve translational relevance, future studies should include aged female mice, ideally ovariectomized, to better model postmenopausal physiology and explore potential sex differences in fractures and associated hepatic responses. Lastly, although this study did not directly examine cellular senescence, it is a hallmark of aging that likely contributes to the elevated baseline inflammation in aged livers. Investigating hepatic senescence-associated markers and evaluating intervention pathways could provide new therapeutic targets for elderly individuals following injury. This is noteworthy given that common metabolic comorbidities in the elderly, such as diabetes and obesity, further suppress SIRT expression and activity, potentially amplifying the age-related deficits described here [78–81]. In summary, the current study lays the foundation for future investigations examining hepatic vulnerability following bone injury. These should ideally incorporate additional timepoints to capture the full inflammatory trajectory, naïve uninjured baseline cohorts, aged female and ovariectomized animals to address sex-specific effects, and fracture models more closely mimicking clinical conditions, such as closed fracture models stabilized with intramedullary nailing, to improve translational relevance.

In conclusion, our data indicates that aged animals exhibit a dysregulated hepatic response to femoral osteotomy, marked by enhanced liver damage, inflammatory cell infiltration, and apoptosis in the context of already elevated inflammation. Despite this pro-inflammatory state, aged mice exhibit blunted activation of RAGE, SIRT1, and JNK pathways, limiting both inflammation resolution and regenerative responses. This imbalance between excessive inflammation and insufficient adaptive signaling may underlie the increased susceptibility of elderly individuals to post-traumatic complications. A deeper understanding of age-related impairments in immune and stress signaling is essential to develop targeted therapies to improve outcomes in the aging population.

## Data Availability

Data are available upon reasonable request from the corresponding author.

## Abbreviations

*AGEs*: Advanced glycation end-products
*BSA*: Bovine serum albumin
*CT*: Comparative threshold-cycle
*CXCL1*: Chemokine (C-X-C motif) ligand 1
*DAMPs*: Damage-associated molecular patterns
*Fx*: Fracture with external fixation
*GAPDH*: Glyceraldehyde 3-phosphate dehydrogenase
*GOT*: Glutamate-oxaloacetate transaminase
*H&E*: Hematoxylin/eosin
*HMGB1*: High-mobility group box 1
*HRP*: Horseradish peroxidase
*IL*: Interleukin
*JNK*: C-Jun N-terminal kinase
*LDH*: Lactate dehydrogenase
*LIS*: Liver injury score
*MAPK*: Mitogen-activated protein kinase
*NAD +*: Nicotinamide adenine dinucleotide
*NE*: Neutrophil elastase
*NF-κB*: Nuclear factor kappa-light-chain-enhancer of activated B cells
RAGE: Receptor for advanced glycation end products
*TNF*: Tumor necrosis factor
